# Delayed Vaccination in Preterm and Low-Birth-Weight Newborns: Predictive Factors and the Impact of Healthcare Professionals’ Knowledge, Attitudes, and Practices

**DOI:** 10.7759/cureus.108643

**Published:** 2026-05-11

**Authors:** Kaoutar Ettoini, Khadija Mesbah, Abdallah Oulmaati

**Affiliations:** 1 Pediatrics, Centre Hospitalier Universitaire Mohammed VI de Tanger, Tangier, MAR

**Keywords:** and practices, attitudes, healthcare professionals, immunization, knowledge, low birth weight, vaccination delay, vaccine hesitancy

## Abstract

Background

Preterm and low-birth-weight newborns are at increased risk of vaccine-preventable infections due to immunological immaturity and reduced transplacental transfer of maternal antibodies. Despite recommendations advocating vaccination according to chronological age, delays in immunization remain frequent in this population, often influenced by healthcare professionals’ knowledge, attitudes, and practices.

Objective

To assess healthcare professionals’ knowledge, attitudes, and practices regarding the vaccination of preterm and low-birth-weight newborns and to identify professional factors associated with delayed vaccination.

Methods

A descriptive and analytical cross-sectional study was conducted in July 2025 among 104 healthcare professionals involved in neonatal care and immunization. Data were collected using a structured, anonymous, self-administered questionnaire assessing knowledge, attitudes, and practices. Statistical analysis was performed using appropriate tests, with a significance level set at p < 0.05.

Results

A total of 104 healthcare professionals participated in the study, of whom 66% had already vaccinated or supervised the vaccination of preterm or low-birth-weight newborns. Significant variability in knowledge and practices was observed across professional categories, particularly between physicians and paramedical staff. Despite prior experience, important knowledge gaps persisted. Four factors were significantly associated with delayed vaccination (p < 0.05): lack of professional training (85%), the belief that prematurity constitutes a contraindication to vaccination (86%), the requirement for a birth weight above 3000 g before initiating vaccination (47%), and parental hesitancy (63%).

Conclusion

Healthcare professionals’ knowledge gaps and inappropriate practices are significantly associated with delayed vaccination in preterm and low-birth-weight newborns. Strengthening targeted training programs and implementing standardized guidelines are essential to improve vaccination practices and ensure optimal protection of this high-risk population.

## Introduction

Preterm and low-birth-weight newborns constitute a particularly vulnerable population to vaccine-preventable infections because of their immunological immaturity and the reduced transplacental transfer of maternal antibodies. For this reason, current recommendations generally advocate vaccination according to chronological age, without correction for gestational age [1,2].

However, several studies have shown that vaccination in these infants remains frequently delayed in clinical practice. This delay appears to be more pronounced among those with lower gestational age and very low birth weight and may also be influenced by organizational and socioeconomic factors [1,3-5].

In addition, healthcare professionals’ attitudes and practices play a key role in the proper implementation of the immunization schedule in this high-risk population. Identifying the factors associated with delayed vaccination and assessing professional practices are therefore essential to improve vaccine coverage among preterm and low-birth-weight newborns [6-9].

The aim of this study was to assess healthcare professionals’ knowledge, attitudes, and practices regarding the vaccination of preterm and low-birth-weight newborns and to identify professional factors that may contribute to delayed vaccination in this population.

## Materials and methods

Study design and setting

A descriptive and analytical cross-sectional study was conducted in July 2025 in Tangier, including healthcare professionals working in primary healthcare centers, maternity units, hospital departments, and neonatal units.

Study objective

To assess healthcare professionals’ knowledge, attitudes, and practices regarding the vaccination of preterm and low-birth-weight newborns and to identify factors associated with delayed vaccination.

Sampling strategy

A non-probability exhaustive consecutive sampling method was used. All eligible healthcare professionals involved in neonatal care or vaccination and present during the study period in selected healthcare facilities, including primary healthcare centers, maternity units, hospital departments, and neonatal units, were approached on-site during working hours and invited to participate. Participation was voluntary, and questionnaires were completed anonymously. The aim was to include all accessible professionals involved in the care, follow-up, counseling, or vaccination of preterm and/or low-birth-weight newborns.

A total of 104 healthcare professionals agreed to participate and completed the questionnaire. Due to the absence of a predefined sampling frame, the response rate could not be precisely calculated.

Inclusion criteria

Healthcare professionals working in primary healthcare centers, maternity units, hospital departments, or neonatal units in Tangier; pediatricians, general practitioners, and nurses; professionals directly involved in the care, follow-up, counseling, or vaccination of newborns and infants; professionals present during the study period and who agreed to participate.

Exclusion criteria

Healthcare professionals absent during the data collection period; professionals not involved in neonatal care or vaccination; refusal to participate; incomplete or unusable questionnaires.

Sample size calculation

The minimum sample size was estimated using the standard formula for cross-sectional studies:

n = (z)2 p ( 1 - p ) / d2

Assuming a 95% confidence level (Z = 1.96), an expected proportion (p) of 50% due to the absence of prior local data, and a margin of error (d) of 10%, the required sample size was 96 participants. After accounting for an estimated 10% non-response rate, the target sample size was approximately 106 participants. A total of 104 healthcare professionals were ultimately included, which was considered adequate and close to the calculated sample size.

Data collection

Data were collected using an anonymous, standardized, self-administered questionnaire assessing sociodemographic characteristics, professional profile, knowledge of vaccination recommendations, attitudes toward vaccine safety and efficacy, and vaccination practices.

The questionnaire was developed based on a review of previously published studies on vaccination practices in preterm infants and structured around the key domains of knowledge, attitudes, and practices (KAP). It was reviewed by two pediatricians and one public health specialist to ensure content validity, clarity, and relevance. A formal pilot testing and psychometric validation, including internal consistency assessment (Cronbach’s alpha), were not performed.

Variables studied

Age, sex, profession, years of experience, workplace, previous training in vaccination, knowledge of the immunization schedule for preterm infants, perceptions regarding vaccine contraindications, self-reported attitudes and practices, and situations associated with delayed vaccination.

Delayed vaccination was defined as any self-reported postponement of vaccination beyond the recommended chronological schedule for preterm and low-birth-weight newborns, according to current immunization guidelines.

Statistical analysis

Data were analyzed using IBM Corp. Released 2022. IBM SPSS Statistics for Windows, Version 28. Armonk, NY: IBM Corp.

Associations between categorical variables were assessed using the Chi-square test or Fisher’s exact test, as appropriate. A binary logistic regression analysis was performed to identify factors associated with delayed vaccination. Results were expressed as odds ratios (ORs) with 95% confidence intervals (95% CI). 

The study variables and questionnaire items are provided in the appendix to facilitate reproducibility.

Variables with a p-value < 0.20 in univariate analysis were considered for inclusion in the multivariate logistic regression model. A p-value < 0.05 was considered statistically significant.

Ethical considerations

Participants’ anonymity and confidentiality were strictly respected throughout the study.

The questionnaire was specifically designed to assess key factors identified in the literature and confirmed in our analysis, including training, misconceptions regarding contraindications, weight-based practices, and parental hesitancy.

## Results

**Figure 1:**
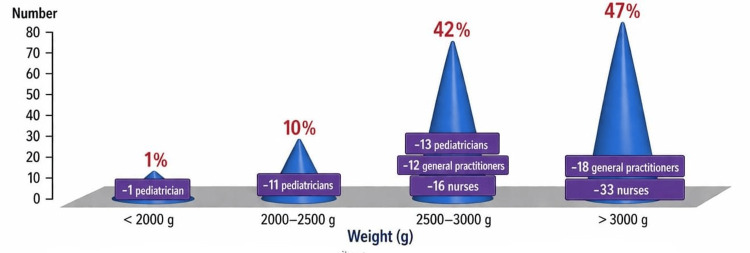
Distribution of healthcare professionals according to the recommended birth weight threshold for initiating vaccination

A total of 104 healthcare professionals participated in our study, of whom 66% had already vaccinated or supervised the vaccination of a preterm or low-birth-weight newborn. The knowledge and attitudes of healthcare professionals are presented in Table 1 and Figure 1. A disparity was observed between pediatricians, general practitioners, and nurses, noting that nurses are primarily responsible for administering vaccinations in first-line practice. A statistically significant correlation (p < 0.05) was identified between four factors and delayed vaccination in preterm infants (Table 2).

**Table 1:** Knowledge and attitudes of healthcare professionals regarding vaccination of preterm and low-birth-weight newborns

Variable	Pediatricians	General Practitioners	Nurses
Total number	25	30	49
Knowledge of specific vaccination schedule for preterm newborns	24	1	4
BCG vaccine is indicated before one month of age in preterm infants	25	3	11
Hepatitis B vaccination in preterm infants depends on maternal vaccination status	25	2	5

**Table 2:** Predictive factors of delayed vaccination in preterm and low-birth-weight newborns

Predictive factors of delayed vaccination in preterm and low-birth-weight newborns	N (%)	Significance level
Lack of training of healthcare professionals in vaccination of this population	88 (85%)	p < 0.05
Belief that prematurity is a contraindication to vaccination	90 (86%)	p < 0.05
Requirement that the newborn reaches a weight above 3000 g before vaccination	49 (47%)	p < 0.05
Parental hesitancy	66 (63%)	p < 0.05

## Discussion

Vaccination of preterm and low-birth-weight newborns remains a major public health priority due to their increased susceptibility to vaccine-preventable infections. Despite clear international recommendations advocating vaccination according to chronological age, delays in immunization continue to be reported in this vulnerable population [1-3]. These delays are multifactorial and involve clinical, organizational, and healthcare professional-related factors [3-5].

In this context, healthcare professionals play a key role in ensuring adherence to vaccination schedules. Their knowledge, attitudes, and practices are associated with vaccination timing and coverage among preterm infants [6-9]. Inadequate knowledge or misconceptions regarding vaccine safety and indications may be associated with unjustified delays, thereby increasing the risk of underimmunization [2].

In our study, a notable variability in knowledge and practices was observed across different professional categories. Similar findings have been reported by Napolitano et al. [6], highlighting the importance of continuous training and access to updated guidelines.

Furthermore, our study identified a statistically significant association between certain professional factors and delayed vaccination. This finding is consistent with previous studies identifying healthcare-related factors as key determinants of immunization timeliness [1,3,4].

Another important observation is the persistence of knowledge gaps despite frequent practical involvement in vaccinating preterm infants. This paradox has also been described in the literature and suggests that clinical experience alone is insufficient to ensure adherence to recommendations [7-11].

Misinterpretation of contraindications represents another major contributor to delayed vaccination. In our study, some healthcare professionals appeared to incorrectly delay vaccination based on factors such as low birth weight or clinical instability, as previously reported [2,12,13].

From an epidemiological perspective, our findings are consistent with studies showing that delayed vaccination is more frequent among infants with lower gestational age and birth weight [1,3,14].

The consequences of delayed vaccination are clinically significant, increasing the risk of insufficient immune protection during a critical period of vulnerability [15,16].

Healthcare professionals, therefore, play a key role in improving vaccination coverage. Targeted interventions, including structured training programs and standardized clinical protocols, have been shown to improve vaccination practices and timeliness [10,11].

Our findings highlight the need to strengthen strategies aimed at improving professional practices. The implementation of standardized protocols, dissemination of simplified guidelines, and regular training programs appear essential to reduce avoidable vaccination delays [10-17].

Finally, delayed vaccination remains a multifactorial issue, with organizational and socioeconomic factors also contributing to delays [4,5].

This study has several limitations that should be acknowledged. First, the cross-sectional design does not allow for the establishment of causal relationships between the identified factors and delayed vaccination. Second, the study was conducted in a single geographic setting, which may limit the generalizability of the findings to other regions or healthcare systems. In addition, the data were based on self-reported responses collected through a questionnaire, which may introduce recall, reporting, and measurement bias, as participants may overestimate or underestimate their actual practices. Furthermore, the assessment of delayed vaccination relied on participants’ reported practices rather than objective clinical records, which may not fully reflect real-world practices. The use of a non-probability consecutive sampling method, the absence of formal psychometric validation of the questionnaire, and the inability to calculate the response rate may also limit the reproducibility and generalizability of the findings. Despite these limitations, this study provides valuable insights into modifiable factors influencing vaccination practices among healthcare professionals.

This study has several strengths. It addresses a clinically relevant and underexplored issue in a high-risk population, namely, delayed vaccination in preterm and low-birth-weight newborns. The inclusion of multiple categories of healthcare professionals allowed for meaningful comparisons across roles and provided a comprehensive overview of knowledge, attitudes, and practices. In addition, the analytical approach enabled the identification of modifiable factors, such as training gaps and misconceptions, which may inform targeted interventions to improve vaccination practices.

## Conclusions

This study highlights significant gaps in healthcare professionals’ knowledge, attitudes, and practices regarding the vaccination of preterm and low-birth-weight newborns. Despite frequent involvement in vaccination, important misconceptions persist, particularly regarding contraindications and vaccination criteria.

Several professional factors were significantly associated with delayed vaccination, including lack of training, erroneous beliefs about prematurity as a contraindication, inappropriate weight-based criteria, and parental hesitancy. These findings suggest a potential role of healthcare providers in vaccination delays in this high-risk population.

Given the well-established benefits and safety of vaccination in preterm infants and the risks associated with delayed immunization, improving professional practices may help reduce delays in vaccination. Strengthening targeted training programs, promoting evidence-based guidelines, and standardizing vaccination protocols may contribute to optimizing vaccination coverage. Ultimately, addressing these gaps may help improve the timeliness of immunization and health outcomes in preterm and low-birth-weight newborns.

## Appendices

Questionnaire used in the study

Section 1: Professional Characteristics

Age: ______

Sex: ☐ Male ☐ Female

Profession: ☐ Pediatrician ☐ General Practitioner ☐ Nurse

Years of experience: ______

Workplace: ☐ Primary care ☐ Maternity ☐ Hospital ☐ Neonatal unit

Section 2: Knowledge

Are you aware of the specific vaccination schedule for preterm newborns? ☐ Yes ☐ No

BCG vaccine is indicated before one month of age in preterm infants: ☐ Yes ☐ No

Hepatitis B vaccination in preterm infants depends on maternal status: ☐ Yes ☐ No

Section 3: Beliefs and Training

Have you received specific training on vaccination of preterm and low-birth-weight newborns? ☐ Yes ☐ No

Do you think prematurity is a contraindication to vaccination? ☐ Yes ☐ No

Do you think parental hesitancy contributes to delayed vaccination? ☐ Yes ☐ No

Section 4: Practice

According to your practice, at what birth weight do you usually initiate vaccination in preterm or low-birth-weight newborns?

☐ < 2000 g

☐ 2000-2500 g

☐ 2500-3000 g

☐ > 3000 g

Do you delay vaccination in preterm or low-birth-weight newborns? ☐ Yes ☐ No

If yes, what are the main reasons for delaying vaccination? (multiple answers possible)

☐ Lack of training in vaccination of this population

☐ Belief that prematurity is a contraindication

☐ Waiting until the newborn reaches a weight >3000 g

☐ Parental hesitancy

☐ Other: ______
